# A case report on urethral prolapse

**DOI:** 10.1093/omcr/omaf216

**Published:** 2025-10-29

**Authors:** Kehinde Awodele, Sunday Charles Adeyemo, Godwin Iyanuoluwa Oyewumi, Olufemi Ebenezer Abidoye, Johnson O Komolafe, Adeniyi Olanipekun Fasanu, Samuel Oluwabunmi Omopariola, Ayodeji Olaolu Oyeniran, Johnson Adekeye Olaore, Olufemi Olamakinwa Ala, Eniola Dorcas Olabode, Ayodele Raphael Ajayi

**Affiliations:** Department of Obstetrics and Gynaecology, Osun State University, P.M.B. 4494, Oke Baale Road. Osogbo, Osun, 230284, Nigeria; Health, society and Well-being, University of Wolverhampton, Wulfruna Street, Wolverhampton, West Midlands, WV1 1LY, United Kingdom; Department of Obstetrics and Gynaecology, Osun State University, P.M.B. 4494, Oke Baale Road. Osogbo, Osun, 230284, Nigeria; Department of Obstetrics and Gynaecology, East Kent Hospitals University NHS Foundation Trust, Kent And Canterbury Hospital, Ethelbert Road, Canterbury, Kent CT1 3NG, United Kingdom; Department of Obstetrics and Gynaecology, Osun State University, P.M.B. 4494, Oke Baale Road. Osogbo, Osun, 230284, Nigeria; Department of Obstetrics and Gynaecology, Osun State University, P.M.B. 4494, Oke Baale Road. Osogbo, Osun, 230284, Nigeria; Department of Obstetrics and Gynaecology, Obafemi Awolowo Teaching Hospital Complex, P.M.B. 5538, Ilesa Road, Ile-Ife, Osun State, Nigeria; Department of Obstetrics and Gynaecology, Osun State University, P.M.B. 4494, Oke Baale Road. Osogbo, Osun, 230284, Nigeria; Department of Obstetrics and Gynaecology, Osun State University, P.M.B. 4494, Oke Baale Road. Osogbo, Osun, 230284, Nigeria; Department of Obstetrics and Gynaecology, Osun State University, P.M.B. 4494, Oke Baale Road. Osogbo, Osun, 230284, Nigeria; Department of Community Health, Obafemi Awolowo University, Ile-Ife, P.M.B. 13. Ile-Ife Osun 220282, Nigeria; Department of Psychiatry medicine, Afe Babalola University Teaching Hospital, Olusegun Obasanjo Way, Ado Ekiti 360102, Ekiti, Nigeria

**Keywords:** urethral, prepubertal, prolapse, girls, protrusion

## Abstract

Urethral prolapse is a rare and often underdiagnosed condition characterized by circumferential eversion of the distal urethra through the external urethral meatus, forming a doughnut-shaped protrusion. We present the case of a 34-month-old girl who was brought to the pediatric emergency unit by her parents following the discovery of bloodstains on her underwear. Clinical examination revealed a reddish, fleshy, doughnut-shaped mass measuring approximately 2.0 × 1.5 cm surrounding the urethral meatus, located above the vaginal introitus and beneath the clitoral hood. She was managed conservatively with oral cefixime suspension (4 mg/kg/day), ibuprofen, sitz baths, and topical estrogen cream. By the fourth day, the bleeding had completely resolved. The prolapsed mass was significantly reduced in size after two weeks and resolved entirely by the sixth week, without residual symptoms. This case highlights the importance of considering urethral prolapse as a differential diagnosis in cases with vaginal bleeding in prepubertal girls.

## Introduction

Urethral prolapse (UP) is an uncommon urogenital condition characterized by circumferential eversion of the distal urethra through the external urethral meatus, forming a doughnut-like protrusion [1]. It predominantly affects prepubertal girls, accounting for over 80% of reported cases. This condition is less frequently encountered in adults, particularly postmenopausal women, the condition is less frequently encountered [2]. Although benign, the rarity and distinctive clinical features of UP can lead to misdiagnosis, especially in populations where it is seldom observed [3]. This has contributed to underreporting across various regions. For example, a five-year review at the Federal Medical Centre, Yenagoa, South–South Nigeria, reported a prevalence of 0.12% of urethral mucosal prolapse (UMP) [4].

The pathophysiology of UP is not fully understood. It is believed to result from a combination of congenital and acquired factors, including collagen deficiency, weak connective tissue attachments within the urethra, increased intra-abdominal pressure, and low estrogen levels in prepubertal girls [5].

Common clinical features include vaginal bleeding, nocturia, dysuria, hematuria, and urinary urgency and frequency. These may mimic urinary tract infections (UTIs) and other pathologies [1].

Management depends on severity and may range from conservative approaches, such as topical estrogen cream, to surgical intervention, if conservative measures fail or complications arise [6].

## Case report

A 34-month-old girl was brought to the pediatric emergency unit by her parents, Yoruba Christians, because of bloodstains observed on her underwear. Bleeding was first noticed five hours prior to presentation, following her return from her first day at school. Despite a change in clothing, staining recurred. There had been no history of previous episodes, genital trauma, insertion of foreign objects, or signs of forceful penetration.

The patient did not report any symptoms of dysuria, urinary frequency, or vulvovaginal itching. There was no family or personal history of bleeding disorders. Her birth and developmental milestones were within the normal limits. She was well nourished, fully immunized, and lived with her employed educated parents in a modest apartment.

**Figure 1:**
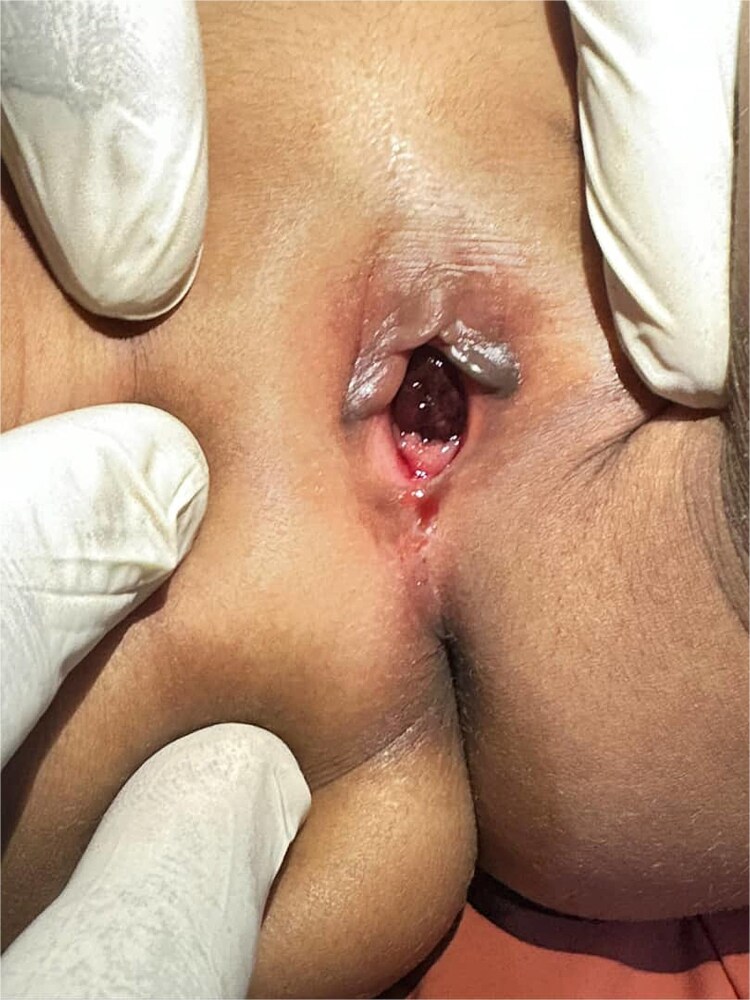
Urethral prolapse presenting as a reddish, doughnut-shaped mass around the urethral meatus in a young female.

Due to concerns about possible sexual abuse, the parents contacted the school and police. A police officer accompanied them to the hospital for the evaluation. On examination, the child was calm, well-nourished, and hemodynamically stable. The systemic examination results were unremarkable. Genital examination revealed a reddish, doughnut-shaped, tender mass (2.0 × 1.5 cm) encircling the urethral meatus, which bled on contact. The hymen were intact with no signs of trauma or foreign bodies (Fig. 1).

The examinations were conducted in the presence of reassured parents. The findings were clearly explained to the parents and attending police officer, emphasizing that the presentation was consistent with urethral prolapse and not indicative of a sexual assault.

Laboratory investigations, including full blood count, urinalysis, urine microscopy, culture, and sensitivity, were all within normal limits.

The patient was commenced on conservative management, including:

Oral cefixime suspension (4 mg/kg/day) to prevent secondary infection.

Ibuprofen suspension for pain and inflammation.

Sitz baths three times daily to relieve edema and maintain hygiene.

Topical estrogen cream applied to the affected area three times daily.

The parents were counseled about the importance of perineal hygiene and adherence to the treatment regimen. They were also educated on the benign nature of their condition and expected course of recovery.

**Figure 2:**
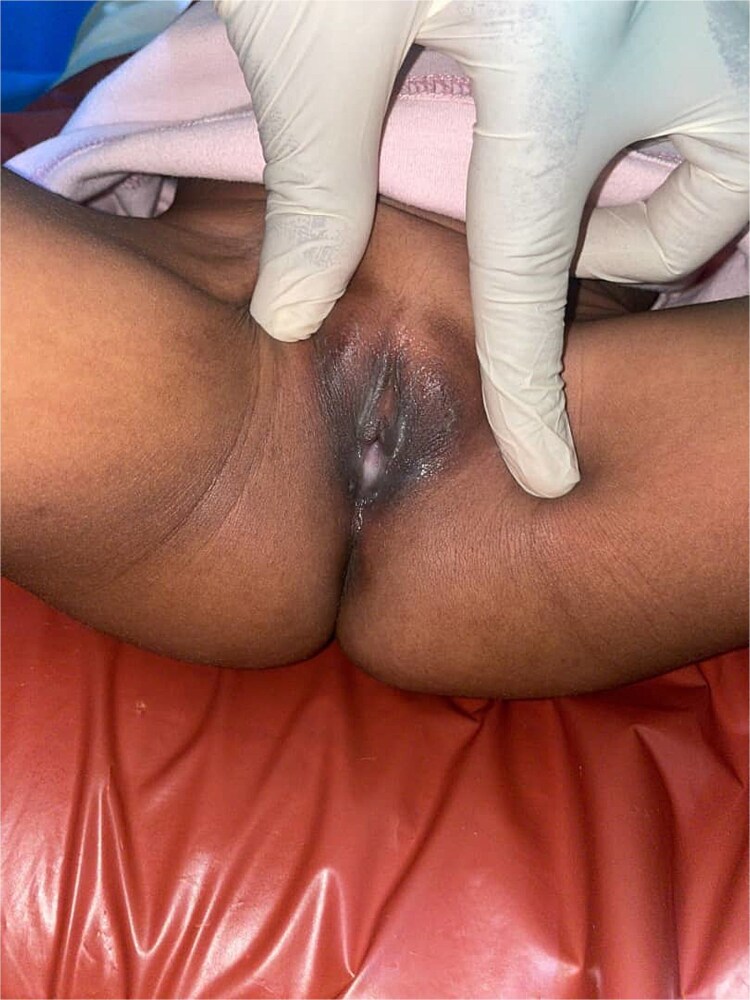
Post-treatment resolution of urethral prolapse, showing normal urethral meatus.

By the fourth day of treatment, the vaginal bleeding had completely resolved. At the two-week follow-up, a marked reduction in the size of the prolapsed mass was observed. By the sixth week, the condition had fully resolved with no residual symptoms or recurrence (Fig. 2).

## Discussion

This case involved a 34-month-old girl who presented with unexplained vaginal bleeding, which initially raised concerns about sexual abuse. However, clinical evaluation confirmed a periurethral mass and a diagnosis of urethral prolapse was established. Although benign, UP is frequently misinterpreted, especially in young girls, owing to its rare presentation and overlap with signs of trauma. Several studies, including those by Mayala et al. [7] and Schaul and Schwark [1], have emphasized that UP can mimic signs of abuse, reinforcing the need for an accurate diagnosis to avoid unnecessary legal action and psychological distress.

In this case, a conservative management approach was effective. The combination of sitz baths, oral antibiotics, NSAIDs, and topical estrogen therapy resulted in rapid symptom resolution and complete recovery within six weeks. Similar outcomes have been reported in literature. Kacimi et al. [8] and Seck et al. [9] documented successful management of pediatric UP using nonsurgical methods.

This report underscores the importance of maintaining a high index of suspicion of UP in prepubertal girls with genital bleeding. Early recognition and sensitive communication with caregivers are vital to prevent misdiagnosis and ensure appropriate noninvasive treatment with favorable outcomes.
